# Computational Design of gRNAs Targeting Genetic Variants Across HIV-1 Subtypes for CRISPR-Mediated Antiviral Therapy

**DOI:** 10.3389/fcimb.2021.593077

**Published:** 2021-03-09

**Authors:** Cheng-Han Chung, Alexander G. Allen, Andrew Atkins, Robert W. Link, Michael R. Nonnemacher, Will Dampier, Brian Wigdahl

**Affiliations:** ^1^Department of Microbiology and Immunology, Drexel University College of Medicine, Philadelphia, PA, United States; ^2^Center for Molecular Virology and Translational Neuroscience, Institute for Molecular Medicine and Infectious Disease, Drexel University College of Medicine, Philadelphia, PA, United States; ^3^Sidney Kimmel Cancer Center, Thomas Jefferson University, Philadelphia, PA, United States

**Keywords:** human immunodeficiency virus type 1 (HIV-1), CRISPR/Cas9, gene therapy, genetic variation, HIV-1 subtypes, bioinformatics, gRNA design

## Abstract

Clustered regularly interspaced short palindromic repeats (CRISPR)-based HIV-1 genome editing has shown promising outcomes in *in vitro* and *in vivo* viral infection models. However, existing HIV-1 sequence variants have been shown to reduce CRISPR-mediated efficiency and induce viral escape. Two metrics, global patient coverage and global subtype coverage, were used to identify guide RNA (gRNA) sequences that account for this viral diversity from the perspectives of cross-patient and cross-subtype gRNA design, respectively. Computational evaluation using these parameters and over 3.6 million possible 20-bp sequences resulted in nine lead gRNAs, two of which were previously published. This analysis revealed the benefit and necessity of considering all sequence variants for gRNA design. Of the other seven identified novel gRNAs, two were of note as they targeted interesting functional regions. One was a gRNA predicted to induce structural disruption in the nucleocapsid binding site (Ψ), which holds the potential to stop HIV-1 replication during the viral genome packaging process. The other was a reverse transcriptase (RT)-targeting gRNA that was predicted to cleave the subdomain responsible for dNTP incorporation. CRISPR-mediated sequence edits were predicted to occur on critical residues where HIV-1 has been shown to develop resistance against antiretroviral therapy (ART), which may provide additional evolutionary pressure at the DNA level. Given these observations, consideration of broad-spectrum gRNAs and cross-subtype diversity for gRNA design is not only required for the development of generalizable CRISPR-based HIV-1 therapy, but also helps identify optimal target sites.

## Introduction

Human immunodeficiency virus type 1 (HIV-1) has been recognized as the causative agent of acquired immunodeficiency syndrome (AIDS) since 1983. Despite controversial evidence for the identification of viral strains that caused zoonotic transmission, phylogenetic analyses suggest that HIV-1 originated from cross-species transmission of simian immunodeficiency virus (SIV) from non-human primates to humans (Keele et al., 2006; Van Heuverswyn et al., 2007; de Silva et al., 2008). Independent zoonotic transmissions of HIV resulted in distinct lineages of HIV-1 viruses that are termed the M, N, O, and P groups (De Leys et al., 1990; Simon et al., 1998; Roques et al., 2004; Vallari et al., 2011). Group M represents the global HIV-1 pandemic with approximately 38 million people living with HIV-1 (Fauci, 2017; World Health Organization, 2020). Due to the rapid genetic divergence during HIV-1 replication coupled with geographic constraints, distinctive lineages within the group M phylogeny evolved independently. These phylogenetic observations were used to designate a collection of HIV-1 subtypes (Hemelaar, 2012). Currently, the field recognizes nine major subtypes (A, B, C, D, F, G, H, J, and K), 96 distinct circulating recombinant forms (CRFs), and unique recombinant forms (URFs) that lack prevalent transmission (Robertson et al., 2000). The average genetic distance within subtypes falls between 8% and 17% with outliers as high as 30% (Korber et al., 2001). Genetic diversity between subtypes ranges from 17% to 42% and has likely been increasing due to evolving recombinant forms (Rambaut et al., 2001; Abecasis et al., 2009).

HIV-1 sequence variation originates from a lack of proofreading during HIV-1 reverse transcription within infected individuals (Hu and Hughes, 2012). Selection pressure due to host immune responses including cell killing and HIV-1-specific antibodies further impact the level of sequence diversity (Phillips et al., 1991; Wei et al., 2003). Error-prone replication and rapid adaptation to host immunity resulted in higher variation within HIV-1 structural genes while viral enzymes diversified at a lower rate (Yu et al., 2004; Kearney et al., 2009; Zanini et al., 2015). Antiretroviral therapy (ART) effectively reduces plasma viral load to undetectable levels, which largely decreases host diversity within a person (Maldarelli et al., 2007; Kearney et al., 2014). However, the establishment of latency across different cellular and anatomical compartments has made ART insufficient to cure HIV-1 infection (Sturdevant et al., 2015; Barton et al., 2016; Bui et al., 2017; Hosmane et al., 2017). Low levels of viral replication within cellular and anatomical reservoirs have also been shown to increase viral diversity under suppressive ART (Palmer et al., 2008; Dampier et al., 2014; Dampier et al., 2016).

Viral rebound has been found in most clinical studies after ART cessation (Persaud and Luzuriaga, 2014; Shah et al., 2014; Henrich et al., 2017; Colby et al., 2018). The major hurdle for developing an HIV-1 cure has been the integrated provirus within the latent reservoir that evades host immune surveillance and ART. Genome editing using the clustered regularly interspaced short palindromic repeats (CRISPR)-Cas9 system has recently been applied to inactivate HIV-1 replication or excise proviral DNA (Ebina et al., 2013; Hu et al., 2014; Zhu et al., 2015; Yin et al., 2016; Kaminski et al., 2016a; Wang et al., 2016a; Kaminski et al., 2016b; Wang et al., 2016b; Lebbink et al., 2017; Yin et al., 2017; Zhao et al., 2017; Bella et al., 2018; Ophinni et al., 2018; Wang Q. et al., 2018; Wang Z. et al., 2018; Dampier et al., 2018a; Dash et al., 2019; Sullivan et al., 2019; Chung C-H et al., 2020). CRISPR-Cas9 is comprised of a guide RNA (gRNA), a 20-nt RNA sequence designed to specify the genomic target that is contained on a larger structural RNA molecule, and the Cas9 endonuclease. This ribonucleoprotein complex facilitates target-specific gene editing using the gRNA to pair with target DNA (also termed as the protospacer), following the recognition of a protospacer adjacent motif (PAM) (Cong et al., 2013; Jinek et al., 2013). Upon sufficient recognition, Cas9 induces double-strand breaks (DSBs) within the target DNA. DSBs will then facilitate endogenous DNA repair mainly *via* non-homologous end joining (NHEJ) in the absence of donor template. The error-prone process of NHEJ-mediated repair has been shown to introduce insertions/deletions (InDels) that render deleterious effects on target genes.

The gene targeting mechanism of CRISPR mediated by the gRNA sequence and the presence of a PAM site has created the opportunity to edit proviral DNA at the site of interest based on functional domains. The 20-bp gRNA sequence that matches the 20-bps of HIV-1 DNA adjacent to a PAM site has dictated the editing region within the HIV-1 provirus. However, the effects of CRISPR-mediated editing vary due to different selection of HIV-1-targeting sites. CRISPR-mediated HIV-1 inactivation could be ineffective by the selection of target sites that are tolerant to InDels without affecting HIV-1 replication. This variation of CRISPR-mediated HIV-1 inactivation has been found in multiple CRISPR screening studies using gRNAs targeting viral genes spanning the HIV-1 proviral genome (Yin et al., 2016; Wang et al., 2016b). HIV-1 diversity further poses critical challenges to the development of this antiviral strategy. Previous screening studies have shown that certain gRNA-protospacer mismatches reduce CRISPR-mediated editing efficiency (Hsu et al., 2013; Doench et al., 2016). This has indicated that even before CRISPR-mediated editing begins, the presence of mismatches between gRNA sequences and HIV-1 target sites reduce the overall editing efficiency within the existing reservoir. Previous studies have shown that patient-derived molecular clones that contained one or more gRNA-protospacer mismatches within the gag and env gene resulted in escape mutants during long-term culture up to 110 days (Darcis et al., 2019).

Editing efficiency is reduced when mismatches exist between gRNAs and target sequences (Hsu et al., 2013; Doench et al., 2016). However, this has been found to be non-linear across the 20-bp protospacer. Position-specific penalty matrices have been developed to quantify the editing efficiency with the presence of gRNA-target mismatches (Hsu et al., 2013; Doench et al., 2016). Hsu et al. developed the MIT matrix, a 20-number vector to indicate a position-specific penalty regardless of mismatch type (Hsu et al., 2013). The cutting frequency determination (CFD) table, a 16x20 matrix, was later developed to incorporate both position and nucleotide specific mismatch-when assigning an editing efficiency penalty (Doench et al., 2016). We and others have demonstrated that the CFD matrix gives better editing-efficiency predictions than the MIT matrix (Cui et al., 2018; Chung C.-H. et al., 2020). Given this observation, the CFD matrix was selected to estimate editing efficiency between candidate gRNAs and HIV-1 genetic variants. In general, a mismatch at PAM-distal regions was more tolerable than PAM-proximal mismatches. In the context of HIV-1 sequence variants, a candidate gRNA may still induce CRISPR-mediated DSBs at the intended target sites if the mismatches existed at PAM-distal side. The CFD matrix assigns a score between 0 and 1, where 1 represents optimal editing efficiency. We have identified a CFD cutoff score of 0.569 to binarize the chance of inducing DSB using publicly available datasets (Haeussler et al., 2016; Chung C.-H. et al., 2020). A CFD score above 0.569 was predicted to induce target editing 95% of the time (Chung C.-H. et al., 2020).

The overall objective of the present study was to propose optimal target sites across HIV-1 subtypes, as well as computationally identify optimal gRNA sequences within selected target sites that cover most existing genetic variants. These target sites will be ideal candidates for developing a pool of gRNAs for targeting any HIV-1-infected individual as opposed to requiring an individually personalized approach. We hypothesize a truly personalized approach would be resource limiting by the need for safety and efficacy trials for each new construct. To accomplish this, we utilized an exhaustive gRNA search pipeline previously developed using k-mer analysis and a position-specific penalty matrix that describes the penalty for gRNA-target mismatches at different positions (Doench et al., 2016; Dampier et al., 2018a; Sullivan et al., 2019). Using this approach, target sites that were conserved across all major subtypes and CRFs were identified. These regions of low diversity also allowed high coverage of known variants in targeted sites across HIV-1 subtypes with carefully chosen gRNA sequences. These analyses resulted in a list of gRNAs proposed for generalized use across patients infected by different subtypes with high predicted CRISPR-mediated efficiency at HIV-1 replication inactivation and a low predicted occurrence of CRISPR resistance.

## Methods

### HIV-1 Sequence Collection From the LANL Database

HIV-1 sequences were retrieved using the sequence search interface on the LANL HIV-1 sequence database with a specified organism of “HIV-1” in the sequence sample (SSAM) section without other limits to the rest of parameters (as of August 2018). Subtype of sequences was specified in the column designated “Subtype” of SSAM section in the LANL HIV01 sequence database. Minor subtypes, CRFs, URFs, and untyped samples were all categorized into ‘Others’. All sequences were used for subsequent analysis as long as the tested gRNA was fully covered by the HIV-1 sequences across a 20-bp nucleotide stretch followed by the presence of a 3-bp PAM.

### Calculation of Global Subtype Coverage, Global Patient Coverage, and Potential Off-Target Sites in the Human Genome

The Subtype Coverage (SC) of a designated gRNA was defined as the number of HIV-1 sequences that resulted in a CFD score above 0.569 divided by the total number of observed sequences within a subtype. The SC was calculated assuming that each sequence is an independent sample. The Patient Coverage (PC) was defined as the number of HIV-1 sequences that resulted in a CFD score above 0.569 divided by the total number of tested sequences observed in the same patient. The potential off-target site in the human genome was defined by any 20-bp genomic sequences that resulted in a CFD score above 0.569 compared with the 20-bp sequence of a tested gRNA. The Pat_SSID column in the LANL database was used to assign sequences to a single patient. The estimated global prevalence was used to weigh the effect of each subtype or patient in the global coverages.

### Calculation of Sequence Diversity

The sequence diversity in this study was determined by a summation of Shannon entropy over a 20-bp window. The sequence diversity was defined as:

$$-\sum_{i=1}^{20}\sum_{j}p_{i,j}\times {\text{log}}_{2}p_{i,j}$$

where *i* is the nucleotide position within the 20-bp window, j is the nucleotide identity (A, C, G, T), and p is the nucleotide probability (e.g. p_10,A_ represents the probability of A at the 10th bp in a given 20-bp window). Sequence diversity ranges between 0 and 40 bits. A sequence diversity of 40 bits means that every base at every position within the window is random. Conversely, a sequence diversity of 0 bits means that every sequence within this window has converged to one variant. The subtype diversity was calculated separately. The global diversity was calculated by the summation of subtype diversity weighted by the estimated global prevalence (Hemelaar et al., 2019).

### Nomenclature of gRNA Identifier

To standardize gRNA names, the 20-bp spacer and corresponding PAM sequence were combined to form a single word used to generate an identifier for each unique gRNA. This conversion was performed by passing the unique gRNA word into the md5 hash function. This generated a 32-letter hexadecimal output. The first six letters from the output were used to generate that corresponding gRNA’s identifier. Within the current number of published gRNAs, the chance of identifier collision should be relatively low.

### Statistical and Bioinformatic Analysis

All bioinformatic analysis was conducted in Python. Simple linear regression analyses were conducted using the scipy.stats package (version=1.4.1). Root-mean-square error was calculated using the sklearn package (version=0.22). Alpha levels were set at 5% for the Wald Tests for the significance of β coefficients (slope) from simple linear regression models. The CRSeek package was used to calculate percent variant coverage and potential off-target cleavage sites adapting cas-offinder (Dampier et al., 2018b).

## Results

### K-Mer-Based Search of Potential gRNAs in the LANL HIV Sequence Database

**Figure 1:**
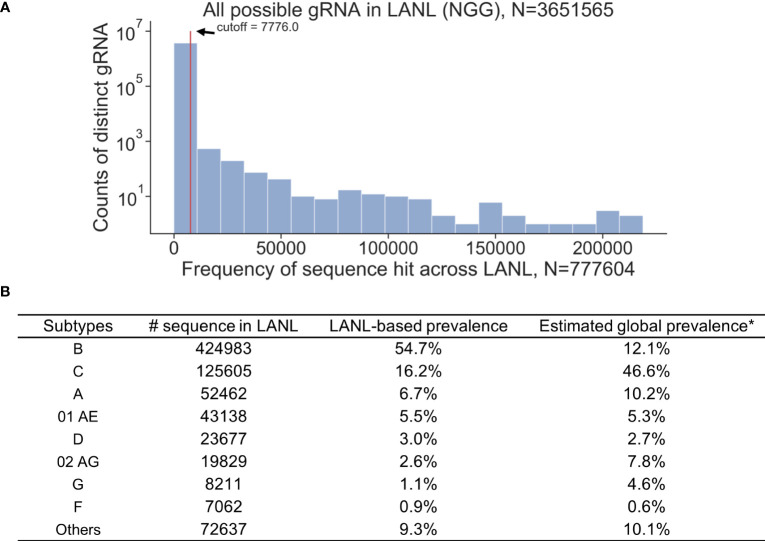
K-mer exhaustive search of all possible distinct 20-bp sequences adjacent to SpCas9 PAM (NGG) using 777,604 HIV-1 sequences across all reported subtypes in the LANL database. **(A)** Distinct 20-bp sequences with high frequencies (seen 1% of time or more; N>7776) in the LANL database were selected as candidate gRNAs. **(B)** The proportion of sequences attributed to each subtype does not represent the global prevalence. Estimated global prevalence was adopted from Hemelaar et al. (2019).

All available HIV-1 sequences deposited in the Los Alamos National Laboratory (LANL) database were used for the analysis (N=777,604, as of August 2018). A 3-bp PAM (NGG, any nucleotide followed by two consecutive guanines) required by Cas9 was the first restriction for gRNA design. An exhaustive search of all 20-mer adjacent to a SpCas9 PAM site were used to collect all possible gRNAs within the LANL database. This resulted in 3,651,565 distinct 20-bp sequences from either sense or antisense strands (**Figure 1A**). However, only 1.9% of possible gRNAs perfectly matched more than 100 sequences within the LANL database. gRNAs that perfectly matched at least 1% (7,776/777,604) of LANL sequences were considered high frequency gRNAs and used for subsequent analysis. Only 1,330 out of 3,651,565 candidate gRNAs exceeded this criterium (**Table S1**). The estimated global editing efficiency of candidate gRNAs was scaled at the population level by the estimated global prevalence for subsequent analysis to account for biased sample size toward subtype B in LANL database (**Figure 1B**). For example, subtype B represents 54.7% of the sequences but only 12.1% of the global prevalence (Hemelaar et al., 2019).

### Cross-Subtype Estimation of gRNA Editing Efficiency

**Figure 2:**
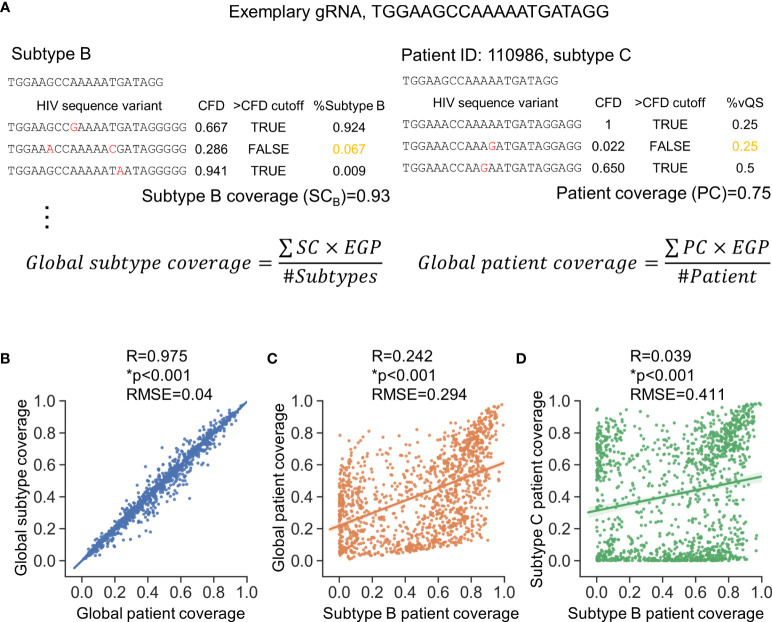
Consideration of global genetic variants is necessary to identify high quality gRNAs. **(A)** Subtype coverage and patient coverage were defined to account for only subtype differences (left panel) or patient-specific differences (right panel), respectively. Subtype coverage was the summation of the frequency of variants that were predicted to be cut (>CFD cutoff) within subtype. Subtype coverage is the unit to account for global subtype coverage. On the other hand, patient coverage represented the summation of frequency of variants identified within patients that were predicted to be cut. The CFD cutoff here is 0.569 where the variants were predicted to be cut when CFD between testing gRNA and variant was larger than 0.569. EGP, Estimated global prevalence; SC, Subtype coverage; PC, Patient coverage. **(B)** Global subtype coverage and global patient coverage was weighted by estimated global prevalence. Both axes represent the percentage of patient infected by specific subtypes that could be treated by a given gRNA. Scale=[0,1]. **(C, D)** The subtype coverage calculated against subtype B is poorly correlated with the global coverage rate **(C)** and the subtype C coverage **(D)**. Correlation coefficient (R) was determined by Pearson correlation; *p<0.001, Wald test was used to test whether variable on the x-axis is a significant predictor of variable on the y-axis; RMSE, root-mean-square error.

Two metrics (shown in **Figure 2A**) were used to assess anti-HIV-1 gRNAs across subtypes; global patient coverage and global subtype coverage. Global patient coverage utilizes data from the *Pat_SSID* column in LANL to group 777,604 sequences by individual “patients.” The coverage for each patient was calculated and then averaged across the database (**Patient coverage section in** **Table S1**). Global Subtype Coverage was calculated by considering each of 777,604 sequences as an independent sample (**Subtype coverage section in** **Table S1**). The global coverage was then calculated by the summation of coverage by each subtype or each patient weighted by the subtype estimated global prevalence (EGP) (**Figure 2A**) (Robertson et al., 2000). The minor variants in global patient coverage is weighted more than that in global subtype coverage, which makes the global patient coverage a suitable metric that accounts for patients possessing only minor variants in latent reservoir for subsequent analyses.

There was a strong positive correlation between global subtype coverage and global patient coverage among the 1,330 candidate gRNAs (**Figure 2B**). Additional correlation tests were conducted to test how subtype coverage of gRNAs designed against Subtype B sequences specified in LANL HIV-1 sequence database, where most research has been conducted, performed against global coverage. This analysis showed only moderate correlation (R^2 =^ 0.242) with a root-mean-squared error of 0.294 (**Figure 2C**). This modest correlation could also be observed between global patient coverage and the subtype-specific patient coverage in all other subtypes (**Figure S1**). Furthermore, inter-subtype patient coverages were even more diverse (**Figure 2D**, **Figure S1**). For example, the root-mean-squared error of patient coverages against subtype B and C among the 1,330 candidate gRNAs was 0.411 (R^2 =^ 0.039), indicating that gRNA design based on one subtype may have limited therapeutic potential for other subtypes.

### Low Diversity Target Sites With High Global Patient Coverage gRNA Sequences Were Identified

**Figure 3:**
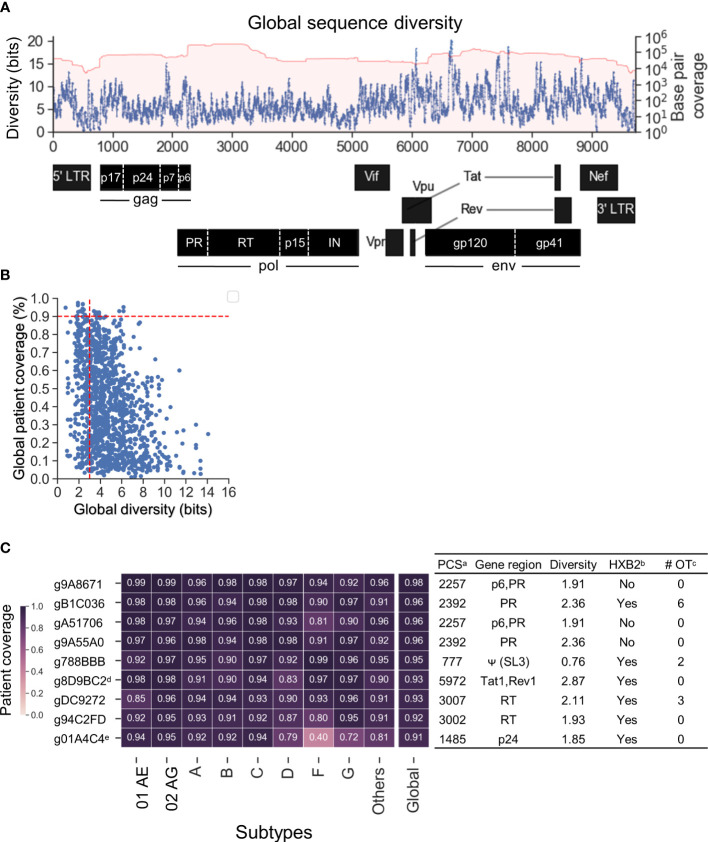
Nine lead gRNAs were identified with high coverage across subtypes. **(A)** The map of global sequence diversity estimated by subtype diversity weighted by estimated global prevalence. **(B)** Scatterplot between global diversity and global patient coverage. Vertical dashed line showed a cutoff of 3 for global diversity and 0.9 for global patient coverage. Nine lead gRNAs were advanced by the cutoff selection. **(C)** The heatmap of subtype-specific patient coverage and global patient coverage. Of the nine gRNAs shown, two were previously published. The additional seven are novel gRNA sequences targeting five distinct HIV-1 genes/motifs as aligned against HXB2. ^a^PCS: Predicted cleavage sites; ^b^HXB2: Whether the absolute gRNA sequence perfectly matched with HXB2 reference sequence. Note that the NL4-3 and R7/E-/eGFP in J-Lat (Chung C.-H. et al., 2020b) also showed the same results as HXB2 (**Table S1**); c# OT: number of potential off-target sites with CFD above 0.569 in human genome. ^d^g8D9BC2: Previously published as gTatRev in (Wang et al., 2016a; Wang et al., 2016b; Darcis et al., 2019); ^e^g01A4C4: Previously published as gGag3 in (Wang et al., 2016a; Zhao et al., 2017).

Previous studies have shown that HIV-1 target sites with low genetic diversity significantly enhanced CRISPR-mediated HIV-1 inactivation efficiency and prevented CRISPR-induced viral escape (Wang et al., 2016a; Wang et al., 2016b; Lebbink et al., 2017; Mefferd et al., 2018). The genomic diversity of each subtype was estimated by calculating the cumulative 20-mer Shannon entropy after the sequences were aligned against HXB2 (Accession number K03455) as described in the *Methods*. The global sequence diversity was the estimated sum of the subtype-specific diversity weighted by the estimated global prevalence shown in **Figure 1B** (**Figure 3A**). The global diversity of target sites among the 1,330 candidate gRNAs ranged between 0.76 and 14.07 bits. Previous studies that have targeted low diversity regions within tat and rev exon 1 (gTatRev, target site:5970-5989) and gag p24 (gGag1, target site: 1389–1408) showed complete viral suppression with no sign of resistance over 110 days of culture (Wang et al., 2016a; Wang et al., 2016b; Darcis et al., 2019). Both were identified in 1,330 candidate gRNAs (g8D9BC2=gTatRev and g80892B=gGag1 in **Table S1**). The global diversity in g8D9BC2 and g80892B was 2.87 and 3.01 respectively (**Table S1**). Candidate gRNAs were also scanned for off-target likelihood (**Table S1**) with representative profiles shown in **Figure S2**.

Given this observation, further gRNA selection criteria were set to a global diversity of 3.01 and global patient coverage to more than 90% (**Figure 3B**). Nine lead gRNAs passed the criteria. There were seven novel gRNAs identified which mainly targeted regions within gag and pol (**Figure 3C**). Two of the nine gRNA sequences, g8D9BC2 (gTatRev) and g01A4C4 (gGag3), have been previously experimentally examined (Wang et al., 2016a; Wang et al., 2016b; Zhao et al., 2017; Darcis et al., 2019). Viral replication was reduced to 2.8% in four independent tests using g8D9BC2 measured by p24 level in the supernatant of culture compared to control. The use of g01A4C4 reduced p24 levels to 5.4% on average in two independent experiments. These observations indicated that the gRNA search method could reproduce previous findings. Note again that this gRNA search method considers both sequence conservation and gRNA-target binding specificity. This means that a highly conserved region is not guaranteed to produce gRNAs with high patient or subtype coverage. For example, although target site 1389–1408 had a low global diversity at 3.01, g80892B (gGag1 in previous studies) was predicted to only cleave 55.5% of patient-derived HIV-1 variants (**Table S1**). More specific, g80892B covered only 52.1% of subtype C variants and 3% of subtype G. Six of nine gRNAs possessed identical sequences to subtype B molecular clones that were commonly used in the literature including HXB2, NL4-3, and R7/E-/eGFP in J-Lat (**Figure 3C**, **Table S1**) (Adachi et al., 1986; St Clair et al., 1991; Jordan et al., 2003). HXB2 was used as the representative for sequence variants that are identical to these reference sequences in subsequent analysis. The number of potential off-target sites was calculated based on the CFD matrix described in the *Methods* section and recorded in **Table S1**. The list of off-target sites and chromosomal positions was illustrated in **Figure S2**.

### Overlapping High-Quality Candidate gRNAs Unraveled the Effect of Absolute Sequence Selection on Consequent CRISPR-Mediated Editing Efficacy

**Figure 4:**
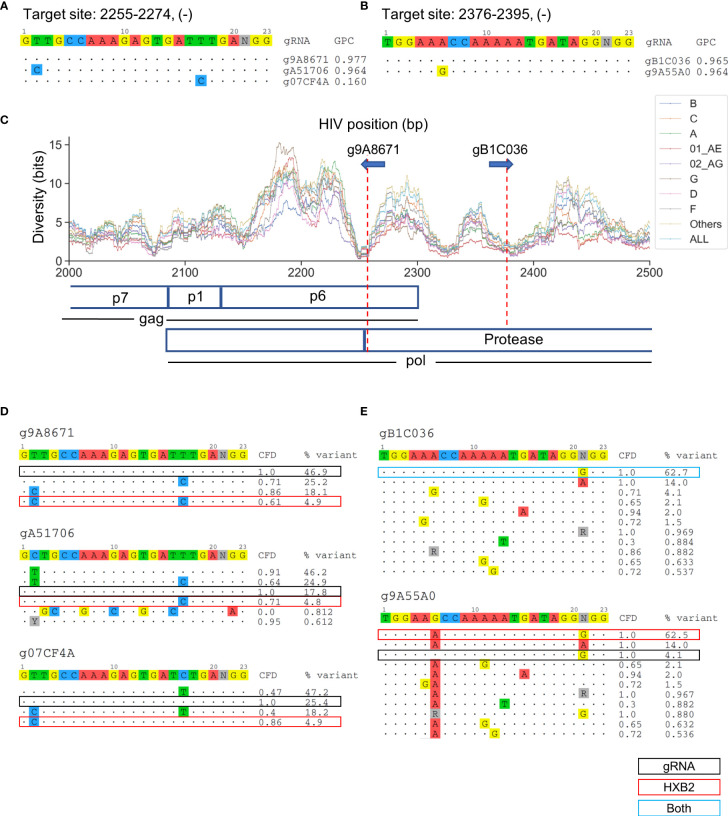
The selection of absolute sequence is likely to affect consequent CRISPR-mediated editing efficacy across sequence variants. **(A)** All candidate gRNAs targeting position 2255–2274 (-) among 1,330 candidate gRNAs. **(B)** All candidate gRNAs targeting position 2376–2395 (-) among 1,330 candidate gRNAs. **(C)** The subtype-specific sequence diversity across the HIV-1 genome between position 2000 and 2500. Both g9A8671 and gB1C036 were predicted to cleave the protease protein in a locally low diversity region. gRNA589 was also predicted to cleave p6. **(D, E)** Sequence variant profiles and corresponding CFD score between chosen gRNA and HIV-1 variants. “HXB2” label next to the % variant represents the variant frequency of the sequence that possessed identical sequence against HXB2.

Overlapping candidate gRNAs were found for individual target gene regions (**Figure 3C**). All candidate gRNAs that overlapped with the nine lead gRNAs on the same target sites were then identified. The target sites g9A8671, gA51706, and g07CF4A overlapped at position 2255–2274 on the HIV-1 genome (**Figure 4A**). They possessed only one bp difference across the 20-bp target region among each other (**Figure 4A**). However, g07CF4A only had 16% global patient coverage (**Table S1**). Another proximal target site at position 2376–2395 had two overlapping gRNAs, g9A55A0 and gB1C036, that differed at position 6 of the 20-bp target site. (**Figure 4B**). Both target sites exhibited high conservation across subtypes (**Figure 4C**). The target site at position 2255–2274 covered both p6 and protease coding sequences, while the other targeted only the protease sequence. To explain these differences, the major sequence variants that represented up to 95% of all known variants were analyzed. g07CF4A held the highest CFD score (0.86) compared to the HXB2 sequence versus g9A8671 (0.61) and gA51706 (0.71) (**Figure 4D**). These scores were all higher than the CFD cutoff of 0.569 used to distinguish whether the gRNA was predicted to induce DSBs 95% of the time (Chung C-H et al., 2020). However, only 4.9% of variants in the LANL database possess an identical target site to HXB2 (**Figure 4D**). This emphasized the advantage of the design method presented here to target the most prevalent genetic variants in the known HIV-1 sequences. In other words, using one reference genome such as HXB2 or NL4-3 is not sufficient to find these gRNAs with high global patient coverage. Using this more generalized design method, gRNAs with high patient coverage could be identified.

Another interesting pair of gRNAs, gB1C036 and gRNA14, were both identified to target position 2376-2395 within HIV-1 protease at high patient coverages (**Figure 4B**). While gB1C036 possessed an identical sequence against the most prevalent variant at this position (62.7%), gRNA14 only perfectly matched to 4.1% of variants in this region (**Figure 4E**). If a gRNA is not predicted to induce DSB on the predominant sequence variants (CFD score below 0.569) it loses the majority of patient coverage. However, the global patient coverage of g9A55A0 (96.4%) remained as high as that of gB1C036 (96.5%) (**Figure 4A**). This could be explained by the nucleotide difference at position six in CFD matrix. The guanine (G) on position six of g9A55A0 did not cause any penalty for the mismatch against adenine (A) on the target DNA. Furthermore, the overall CFD score was increased between g9A55A0 sequence and all major variants (**Figure 4E**).

### Sequences Responsible for the Packaging Signal Are Under High Evolutionary Constraint

**Figure 5:**
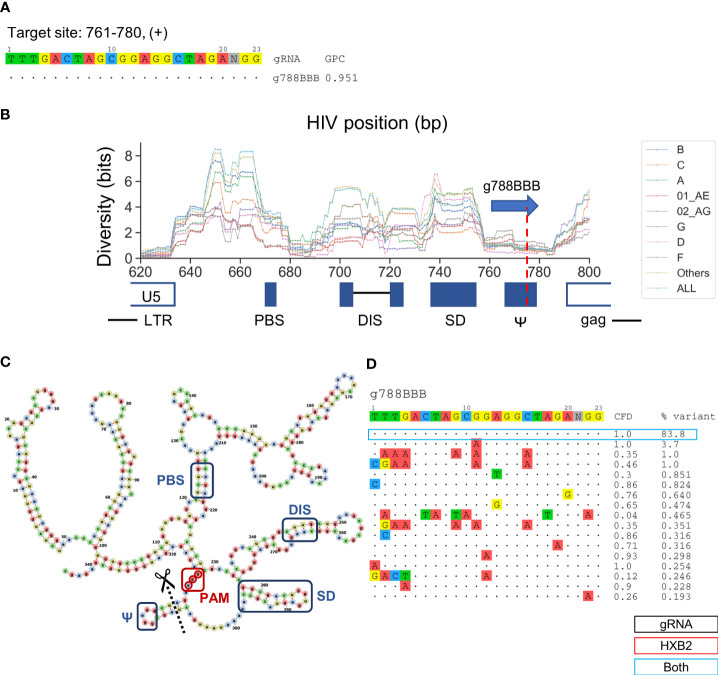
Low diversity in the SL3 region is an ideal target for the selection of gRNA sequence that provides high predicted global patient coverage. **(A)** All candidate gRNAs targeting position 761-780 (+) among 1,330 candidate gRNAs. **(B)** The subtype-specific sequence diversity across the HIV-1 genome between position 600 and 800. The predicated target region showed a local trough flanking by g788BBB target sites. g788BBB was predicted to cleave nucleocapsid binding site, Ψ. **(C)** Predicted HXB2 untranslated region (UTR) secondary structure with the PBS (primer binding site), DIS (dimer initiation site), SD (splicing donor), and Ψ regions labeled with blue, g788BBB PAM labeled with red, and cleavage site labeled with black. Secondary structure was predicted using RNAfold (Gruber et al., 2008) and visualized using VARNA (Darty et al., 2009). **(D)** Sequence variant profiles and corresponding CFD score between chosen gRNA and HIV-1 variants. “HXB2” label next to the % variant represent the variant frequency of the sequence that possessed identical sequence against HXB2.

g788BBB was found to target a sequence region that should inactivate HIV-1 replication but has been previously uninvestigated (**Figure 5A**). This gRNA targets positions 761–780 across a sequence motif that forms one of the four functional RNA stem-loop secondary structures, conventionally termed SL3 (stem-loop 3) or Ψ, for nucleocapsid (p7) to bind during HIV-1 genome packaging (De Guzman et al., 1998). A previous study has showed lower sequence diversity within the SL3 region than adjacent genomic regions, indicating that mutations in this region are rarely tolerated (Ingemarsdotter et al., 2018). The predicted cleavage site was at position 777, which would likely induce InDels that disrupt the stem loop formation (**Figures 5B, C****)**. Furthermore, the PAM site was located 7-bp upstream of the translation initiation site (**Figure 5C**). More than 83.8% of sequences across subtypes were identical within the 20-bp target region, which was in agreement with previous observations. (**Figure 5D**) (Ingemarsdotter et al., 2018). This is the highest percentage of predominant variants covered among the nine lead gRNAs. Most of the minor variants existed in subtype 01 AE, B, and D indicated by the subtype-specific patient coverage (**Figure 3C**).

### The Reverse Transcriptase-Targeting gRNA Covered a Conserved Subdomain Responsible for dNTP Incorporation

**Figure 6:**
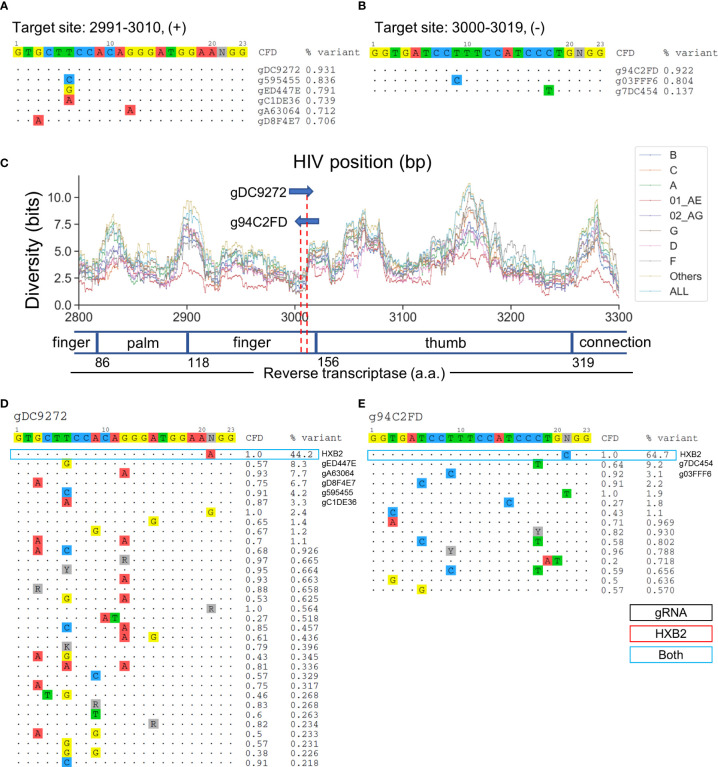
Two lead gRNAs target critical dNTP binding domain in HIV RT protein that is responsible for effective reverse transcription. **(A)** All candidate gRNAs targeting position 2991-3010 (+) among 1,330 candidate gRNAs. **(B)** All candidate gRNAs targeting position 3000-3019 (+) among 1,330 candidate gRNAs. **(C)** The subtype-specific sequence diversity across the HIV-1 genome between position 2800 and 3300. Major domains are shown in the open or closed blocks. The numbers next to domain names indicate the amino acid coordinate for reverse transcriptase. **(D, E)** Sequence variant profiles and corresponding CFD score between chosen gRNA and HIV-1 variants. “HXB2” label next to the %variant represent the variant frequency of the sequence that possessed identical sequence compared with HXB2. The sequence variants labeled for gRNAs represent the gRNA contains the identical sequence with sequence variants.

Target sites at positions 2991-3010 and 3000-3019 cover RT at residues 148-155 and 150-157, respectively (**Figures 6A, B****)**. gDC9272- and g94C2FD-targeted sites reside in the second finger subdomain in RT (**Figure 6C**). Previous studies have shown that the Q151 residue was responsible for direct interaction with the 3’-OH of the incoming dNTP (Huang et al., 1998). The Q151M mutation confers resistance against most nucleoside RT inhibitors (NRTIs), nucleoside analogs that lack the 3’-OH group (Ueno et al., 1995). The predicted cleavage site of g94C2FD was between residues 151–152, while gDC9272 was predicted to cleave between the second and third codon of residue 153 (**Figure 6C**). gDC9272 was predicted to target 26/32 observed sequence variants listed in **Figure 6D**, which allowed for a 93.1% global variant coverage. A C-to-T mismatch at position 18 between the g94C2FD and the sequence variant with 9.2% frequency resulted in moderate reduction of CFD score to 0.64 (**Figure 6E**). However, this reduction was not predicted to prevent CRISPR-mediated editing because the CFD score was still higher than the cutoff.

## Discussion

The overarching goal of this study was to generate broad-spectrum gRNAs for the generalized use of CRISPR-mediated antiviral therapy across patients infected by different subtypes. Multiple sequence alignments can produce spurious noise when aligning sequences with high diversity and numerous recombination events while a k-mer approach is unphased by this noise. In this study, the standard k-mer counting technique was supplemented with CRISPR-mediated editing mechanisms to optimize gRNA design methods. A pool of 20-bp candidate gRNAs that were conserved across sequence variants of all subtypes was selected. The data suggested that the consideration of subtype differences was essential during the design process. The gRNAs designed by incorporating sequence variants within single subtypes did not guarantee effectiveness across other subtypes (**Figure 2D** and **Figure S1**). A similar result was shown that previously proposed anti-HIV-1 gRNAs often lack homology to major sequence variants seen across infected patients (Dampier et al., 2017). The metric used in this study to calculate global patient coverage and subtype coverage accounted for the difference of existing variation between subtypes.

Sequence variant coverage accounted for the likelihood a gRNA sequence that can cause DSBs on the collection of known variants. However, it did not estimate the functional relevance of targeted loci in the HIV-1 lifecycle. Previous studies have used sequence conservation to select HIV-1 target regions (Ebina et al., 2013; Liao et al., 2015; Ueda et al., 2016; Mefferd et al., 2018; Yin et al., 2018). The reduced sequence diversity was a consequence of low tolerance of mutations at functionally important domains. HIV-1 required a longer time to develop resistant strains when the gRNAs targeted more conserved regions (Wang et al., 2016b). A systematic approach that analyzed experimental readouts with the use of anti-HIV-1 gRNAs also demonstrated a positive correlation between functional reduction and sequence diversity at target sites. The low diversity regions identified in this study may point to sequence motifs with significant functions that were preserved in all HIV-1 subtypes. The final list of lead gRNAs was identified at the regions where the broad-spectrum sequence variants and low diversity sequence variants converged. This result made clear that the metrics optimizing absolute gRNA sequence and determining target sites agreed with each other better when the entropy was reduced. Note that three lead gRNAs identified in this study were predicted to have potential off-target cleavage only at intergenic or intron regions. Further validation with respect to off-target cleavage using genome wide sequencing approach similar to previous study as well as *in vitro* cell viability test are required to support the safety of proposed gRNAs (Chung C.-H. et al., 2020a).

The gRNA search method identified nine gRNAs possessing global patient coverage of 0.9 or higher; seven of the novel gRNA sequences that mapped to five distinct target sites have not been previously tested in the literature. Interestingly, the results presented in this study showed that the absolute sequence of gRNA and the target sequences across HIV-1 sequence variants largely affected the outcome of predicted patient coverage. All lead gRNAs were found to be identical to the most predominant variant at the target sites. An interesting result regarding g9A55A0 is that while it contained at least one mismatch to more than 96% of sequences, it still exhibited high patient coverage. It is possible that this phenomenon could be due to lack of experimental results that informed the relationship between this specific pairwise identity when the CFD matrix was derived. Functional studies are warranted to first distinguish whether the G-to-A mismatch at position 6 reduced editing efficiency against HXB2 or other molecular clones that contain the same target site sequence. In addition, this work demonstrated the computational strategy to identify optimal gRNAs targeting sites using the CFD matrix, which was derived from the data using SpCas9 system. This strategy could be adapted by other Cas orthologs. All potential gRNAs targeting HIV-1 LAI reference genome using SaCas9, nmCas9, and Cpf1 were listed in a previous study with little consideration given to HIV-1 genetic variation and subtype differences (Yoder, 2019). The same pipeline developed in this study could be utilized for gRNA screening and selection with different Cas systems if the position-specific penalty matrices are available. However, the CFD matrix was derived from functional study using only SpCas9. This means that a Cas ortholog might have an orthogonal penalty matrix that requires a systematic experimental design in independent studies. For example, a SpCas9 derivative such as SpCas9-HF1 or HiFi-SpCas9 may have similar behavior as SpCas9 but not identical (Kleinstiver et al., 2016; Vakulskas et al., 2018).

SL3 is one of the targetable sites that was previously unexamined in the test of HIV-1 inactivation efficiency using CRISPR/Cas. The 300-bp untranslated region (UTR) of the HIV-1 genome forms secondary RNA structures and plays a crucial role in the HIV-1 replication cycle. The transactivation response (TAR) element at the beginning of transcription has been found to be conserved and effective at reducing HIV-1 replication using TAR-targeting gRNAs (Hu et al., 2014; Kaminski et al., 2016b; Yin et al., 2016; Bella et al., 2018). However, other functional motifs in the UTR were rarely tested using the CRISPR system. A previous study examined gRNA gPBS1-3, which targeted a low diversity region located in primer binding site (PBS). The time of emerged escape mutants was delayed by gPBSs but did not circumvent later onset of resistance (Wang Z. et al., 2018). However, small molecules targeting secondary RNA structure in HIV-1 remain early in the developmental process for clinical use (Warui and Baranger, 2012; Ingemarsdotter et al., 2018). CRISPR-mediated editing is ideal for targeting non-coding functional domains since its mechanism of action is on the DNA level. This indicated a new modality to intervene in non-coding sequence regions that conventional biologics have found difficult to target. The characteristic of Ψ holds great promise for the use of CRISPR-mediated inactivation strategy due to low sequence diversity and reduced number of distinct variants across all subtype present in the LANL database. It indicates that this region has been under high evolutionary pressure to reduce background mutations and to serve as an ideal target for gRNA design.

Overall, the novel target sites with optimal gRNA sequences were identified with promising characteristics of functional importance. Functional assays are warranted to validate the efficiency at reducing HIV-1 replication. A viral swarm derived from HIV-1-infected individuals was also proposed to take closer evaluation with respect to the ability of targeting genetic variants that were naturally occurred within and between patients.

## Data Availability Statement

The HIV-1 sequence datasets for this study can be found in the search interface in the LANL website [https://www.hiv.lanl.gov/components/sequence/HIV/search/search.html]. The analyses and figures can be reproduced in Python notebook (https://github.com/DamLabResources/HIV-subtype-gRNAs).

## Author Contributions

Conceived idea and experimental design—C-HC, AAl, WD, MN, and BW. Data collection—C-HC. Intellectual contribution—AAt, MN, and BW. Prepared manuscript—C-HC and WD. Critical reading and revision—AAl, AAt, RL, MN, WD, and BW. All authors contributed to the article and approved the submitted version.

## Funding

This work was supported by National Institute of Mental Health (NIMH) R01 MH110360 (Contact PI, BW), NIMH Comprehensive NeuroAIDS Center (CNAC) P30 MH092177 (Kamel Khalili, PI; BW, PI of the Drexel subcontract involving the Clinical and Translational Research Support Core, Drexel Component PI, BW), and the Ruth L. Kirschstein National Research Service Award T32 MH079785 (BW, Principal Investigator of the Drexel University College of Medicine component and Dr. Olimpia Meucci as Co-Director). The contents of the paper were solely the responsibility of the authors and do not necessarily represent the official views of the NIH. AAl was also supported by the Drexel University College of Medicine Dean’s Fellowship for Excellence in Collaborative or Themed Research (AAl, fellow; BW, mentor).

## Conflict of Interest

The authors declare that the research was conducted in the absence of any commercial or financial relationships that could be construed as a potential conflict of interest.
