# Supplementary material for: Computational Design of gRNAs Targeting Genetic Variants Across HIV-1 Subtypes for CRISPR-Mediated Antiviral Therapy
Source: Front Cell Infect Microbiol. 2021 Mar 9;11:593077. doi: 10.3389/fcimb.2021.593077 (PMC7985454; doi:10.3389/fcimb.2021.593077)
Supplement: Supplementary file 1 [file DataSheet_1.pdf]

Supplementary

Design of gRNAs targeting genetic variants across HIV-1 subtypes for CRISPR-mediated antiviral therapy

Cheng-Han Chung[1,2], Alexander G. Allen[1,2], Andrew Atkins[1,2], Robert W. Link[1,2], Michael R. Nonnemacher[1,2,3], Will Dampier[1,2], Brian Wigdahl[1,2,3, *]

Table of contents

Figure S1. Pairwise subtype-specific patient coverage across nine selected subtypes and global patient coverage

Figure S2. Three of nine lead gRNAs that were predicted to have any predicted off-target cleavage.

Table S1. List of 1330 candidate gRNAs and corresponding characteristics with respect to target sites, predicted efficiency, local diversity, and predicted off-target sites

Figure S1. Pairwise subtype-specific patient coverage across nine selected subtypes and global patient coverage. Each plot has 1330 data points that represent 1330 candidate gRNAs. Diagonal figures show the histograms of variable distributions. The numbers of gRNA patient coverages could be found in the Patient Coverage columns in Table S1. 'PatCovered' showed the average variant coverages within patients using given gRNA when tested in between patients infected by different HIV-1 subtypes. All data points ranged from 0 to 1, while 1 indicated 100% of observed variants were predicted to be cut by tested gRNA. Patient coverage and global patient coverage were defined in Figure 2A. The subtype-specific patient coverage ([subtype]+PatCovered) was the average patient coverage for all patients infected by defined subtype in LANL HIV-1 sequence database. Diagonal shows the distribution of patient coverage of 1330 gRNAs against each subtype.

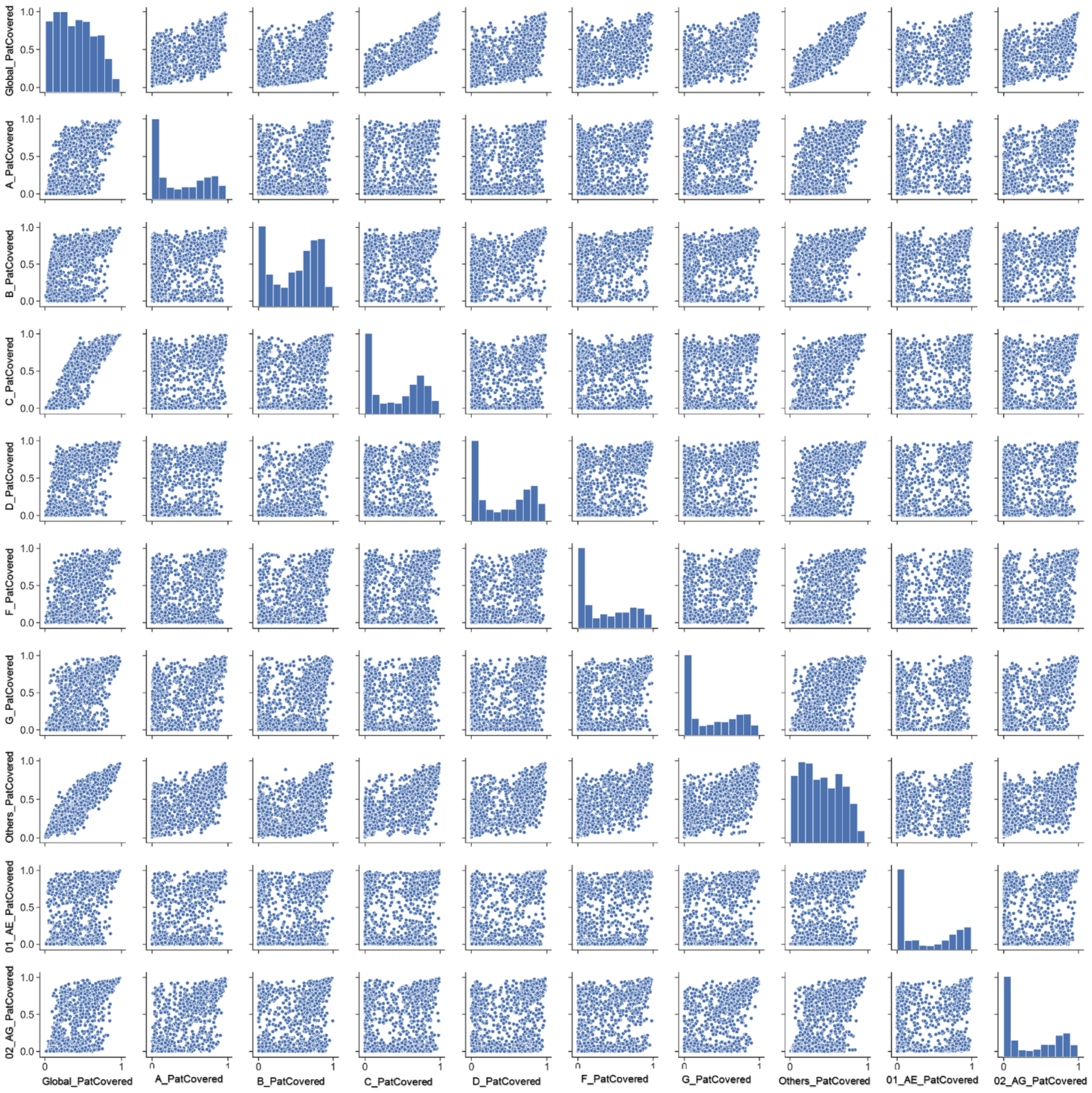

Figure S2. Three of nine lead gRNAs that were predicted to have any predicted off-target cleavage. The position shown indicates the most 5' position to which a 23-bp gRNA would potentially bind. The region shows the gene region the gRNA locates on. The number followed by intron indicates the number of interspaced intron from the begin of transcription for a given gene.

A

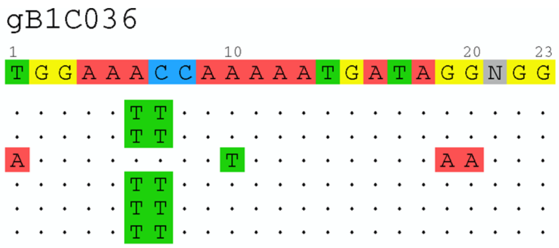

Position	Region
Chr11:71541347	Intergenic
Chr12:8316370	Intergenic
Chr3:172704957	Intron4:SPATA16
Chr4:9399528	Intergenic
Chr8:7797646	Intron2:ZNF705B
Chr8:7229265	Intron2:ZNF705G

B

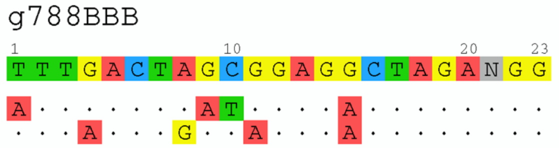

Position	Region
Chr16:22911579	Intron1:HS3ST2
Chr4:139035800	Intron3:LINC00616

C

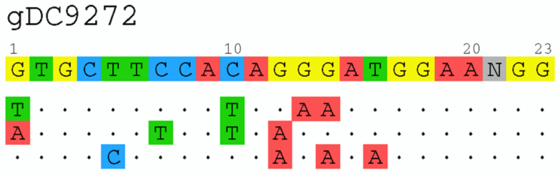

Position	Region
Chr11:59709909	Intergenic
Chr8:25796128	Intron6:EBF2
Chr9:119607641	Intron11:ASTN2

Tables S1. List of 1330 candidate gRNAs and corresponding characteristics with respect to target sites, predicted efficiency, local diversity, and predicted off-target sites (Available for download). Column 'spacer' is the 20-bp RNA sequence of the gRNAs without the PAM sequence for the input of CRSeek computational tool (1). Column 'g5to3' is the gRNA entry sequence for the MD5 hash conversion with PAM sequence locating at the 3' end. The 32-letter in hexadecimal is recorded in column 'hex_full', while the first six letters of the 32-letter string was recorded in column 'hex' and used for comparison purposed. The name of gRNAs presented in this chapter adds letter 'g' in front of capitalized hex identifier and shows in column 'name'. Column 'strand' shows the corresponding orientation against HXB2 reference sequence (Accession number K03455). The start and stop sites are the 20-bp target sites where the gRNA locates on the HIV-1 genome based on the HXB2 coordinate. The cleavage site is the potential position of CRISPR-induced DSB loci when using corresponding gRNAs. Column 'Region' shows the HIV-1 genes the gRNA overlaps with at least one bp. The parameters including the average CFD score, subtype coverage, patient coverage and sequence diversity were first calculated by subtype-specific sequences overlapping with the designated target sites. Each subtype-specific score was weighted to calculate the global effect of each parameter. Column 'offcount' shows the predicted number of potential off-target sites in the human genome (hg19) with a CFD score above 0.569, the CFD cutoff determined in (2), when using the designated gRNAs in human cells. Column 'inPubs' shows whether the sequence of candidate gRNA has been published in the literature using the hex identifier mentioned above. Columns 'inHXB2', 'inJLat', and 'inNL43' indicate whether the sequence of the candidate gRNA is identical to the reference genome HXB2, integrated virus in J-Lat 10.6 cells, or NL4-3 molecular clone, respectively.

References:

1. Dampier W, CH Chung, NT Sullivan, A Atkins, MR Nonnemacher, and B Wigdahl. CRSeek: a Python module for facilitating complicated CRISPR design strategies. PeerJ 2018.
2. Chung C-H, AG Allen, AJ Atkins, NT Sullivan, G Homan, R Costello, R Madrid, MR Nonnemacher, W Dampier, and B Wigdahl. Safe CRISPR/Cas9 inactivation of HIV-1 transcription with high specificity and broad-spectrum activity in latently infected cells by mutation of HIV-1 promoter NF-κB binding sites. Molecular Therapy-Nucleic Acids 2020.
